# Exosome-Based Delivery of Super-Repressor IκBα Alleviates Alcohol-Associated Liver Injury in Mice

**DOI:** 10.3390/pharmaceutics15020636

**Published:** 2023-02-14

**Authors:** Hee-Hoon Kim, Young-Ri Shim, Sung Eun Choi, Tolulope Esther Falana, Jae-Kwang Yoo, So-Hee Ahn, Minhye Park, Hyangmi Seo, Chulhee Choi, Won-Il Jeong

**Affiliations:** 1 Laboratory of Liver Research, Graduate School of Medical Science and Engineering, KAIST, Daejeon 34141, Republic of Korea; 2 ILIAS Biologics Inc., Daejeon 34014, Republic of Korea

**Keywords:** alcohol-associated liver injury, exosome, nuclear factor-κB, inflammation, Kupffer cell

## Abstract

Activation of Kupffer cells (KCs) by gut-derived lipopolysaccharide (LPS) instigates nuclear factor-κB (NF-κB)-mediated inflammatory responses in alcohol-associated liver diseases (ALD). Here, we utilized a novel optogenetically engineered exosome technology called ‘exosomes for protein loading via optically reversible protein–protein interactions (EXPLOR)’ to efficiently deliver the super-repressor IκB-loaded exosomes (Exo-srIκB) to the liver and examined its therapeutic potential in acute-on-chronic alcohol-associated liver injury. We detected enhanced uptake of DiI-labeled Exo-srIκB by LPS-treated inflammatory KCs, which suppressed LPS-induced inflammatory gene expression levels. In animal experiments, a single intravenous injection of Exo-srIκB prior to alcohol binge drinking significantly attenuated alcohol-associated hepatic steatosis and infiltration of neutrophils and macrophages but not a liver injury. Notably, three consecutive days of Exo-srIκB injection remarkably reduced alcohol-associated liver injury, steatosis, apoptosis of hepatocytes, fibrosis-related gene expression levels in hepatic stellate cells, infiltration of neutrophils and macrophages, and inflammatory gene expression levels in hepatocytes and KCs. In particular, the above effects occurred with inhibition of nuclear translocation of NF-κB in liver tissues, and these beneficial effects of Exo-srIκB on ALD were shown regardless of doses. Our results suggest an exosome-based modulation of NF-κB activity in KCs by Exo-srIκB as a novel and efficient therapeutic approach in ALD.

## 1. Introduction

Alcohol-associated liver disease (ALD) caused by chronic and excessive alcohol consumption is one of the most prevalent chronic liver diseases worldwide [1]. Usually, ALD follows a well-defined disease spectrum starting with the alcohol-associated fatty liver (AFL) that develops into alcohol-associated steatohepatitis, fibrosis (ALF), cirrhosis (ALC), and hepatocellular carcinoma (HCC) [2]. Chronic alcohol consumption induces gut dysbiosis and increases intestinal permeability, resulting in the hepatic influx of abnormally high amounts of pathogen-associated molecular patterns, including lipopolysaccharide (LPS), through the portal circulation [1]. Although Kupffer cells (KCs) take up and detoxify gut-derived LPS under the homeostatic condition [3], elevated levels of LPS by chronic alcohol consumption are sensed by toll-like receptor 4 (TLR4) on KCs and initiate alcohol-associated hepatic inflammation. In particular, TLR4-mediated activation of nuclear factor-κB (NF-κB) triggers the production of pro-inflammatory cytokines and chemokines such as tumor necrosis factor (TNF)-α, interleukin (IL)-6, pro-IL-1β, C-C motif chemokine ligand 2 (CCL2), and C-X-C motif chemokine ligand 1 (CXCL1) by KCs and accelerates the progression of ALD [1,4,5]. Therefore, targeting NF-κB signaling in KCs may be a viable therapeutic approach in ALD.

The LPS-mediated NF-κB signaling activation involves the transfer of LPS by LPS-binding protein (LBP) to a cluster of differentiation 14 (CD14), followed by binding with the TLR4/myeloid differentiation factor 2 complex. Oligomerization of TLR4 triggers recruitment of downstream adaptors to myeloid differentiation primary response gene 88 (MyD88), resulting in the consequential activation of transforming growth factor β-activated kinase 1 (TAK-1) [6]. In ALD, TAK-1 activation provokes the release of NF-κB and its nuclear translocation via IκB kinase (IKK)-mediated phosphorylation of IκB, unlike the homeostatic state in which IκB binds to NF-κB in the cytosol and restricts its nuclear translocation [7]. It has been reported that mice deficient with LBP [8], CD14 [9], or TLR4 [10] showed decreased alcohol-associated liver injury (ALI) compared to wild-type (WT) controls. The above promising results from animal experiments prompted the discovery or synthesis of therapeutic agents that suppress NF-κB activation for clinical usage in ALD. Indeed, more than 700 NF-κB inhibitors that target various stages of the NF-κB signaling pathway have been developed [11], and some of them have commenced clinical trials for inflammatory liver diseases, including autoimmune hepatitis, non-alcoholic steatohepatitis, and chronic hepatitis C viral infection [12]. Despite these efforts, however, there are no approved NF-κB blockers and therapeutics for ALD except liver transplantation or abstinence.

An impediment to the drug development is the successful and efficient delivery of drugs to target cells to minimize adverse effects. Nanoparticles have been highlighted for the targeted delivery of small molecules, nucleic acids, and proteins [13]. In particular, galactose-coated nanoparticles target hepatocytes (HEPs), which exclusively express the asialoglycoprotein receptor [14]. However, it has been well known that the reticuloendothelial system-mediated clearance limits the target cell delivery of nanoparticles [15]. Therefore, instead, exosomes have attracted attention as a novel drug carrier system. Exosomes are extracellular vesicles that are between 40 and 120 nm in size and can be generated and secreted by the various cell types in our body. The biological function of exosomes is to mediate intercellular communication by delivering diverse cargo, including nucleic acids, proteins, and lipids [16]. Exosome-based drug delivery provides many advantages since it has a high capacity to store drugs, overcome biological barriers, and target specificity [17]. Recently, a novel, optogenetically engineered exosome technology called ‘exosomes for protein loading via optically reversible protein–protein interactions (EXPLOR)’ has been developed that enhances the biological compatibility and production efficiency of exosomes [18]. In addition, by utilizing EXPLORs, Yim et al. engineered exosomes to load IκB protein without IKK phosphorylation sites called ‘super-repressor IκB’ (Exo-srIκB) that has a prolonged half-life and sustained inhibition of NF-κB [18]. Follow-up studies with Exo-srIκB have demonstrated that in vivo delivery of Exo-srIκB attenuates sepsis-associated organ damage and mortality, inflammatory responses related to preterm birth, and ischemia-reperfusion kidney injury in mice [19,20,21]. In addition, in vivo tracing of the biodistribution of zirconium-89 (^89^Zr)-labeled Exo-srIκB in mice revealed a predominant delivery of Exo-srIκB to the liver after intravenous injection [22].

Based on the above findings, here, we aimed to assess the protective effects of Exo-srIκB on ALI. Since there is no approved drug for ALD except abstinence or liver transplantation [1], the development of novel therapies is urgently needed. In this aspect, our findings may advance the field of ALD by providing Exo-srIκB as a useful therapeutic agent.

## 2. Materials and Methods

### 2.1. Mice

C57BL/6J WT male mice (Jackson Laboratory, Bar Harbor, ME, USA) aged 8 to 10 weeks (about 25 g of body weight) were used for in vivo experiments. All in vivo studies were approved by the Institutional Animal Care and Use Committee of the Korea Advanced Institute of Science and Technology (KAIST) (Approved No. KA2021-071). Mice were maintained in a specific pathogen-free facility at KAIST with a standard 12 h light and dark cycle. There was no bias for the location, humidity, and temperature of cages. Mice were acclimatized with feeding location for at least 1 week and with Lieber–DeCarli ethanol (EtOH) diet (Dyets Inc., Bethlehem, PA, USA) for 5 days. After that, the percentage of EtOH in the diet was gradually increased (1 to 5%) for another 5 days. After a total of 17 days of acclimatization periods, mice were fed with 5% EtOH-containing diet for 10 days [23]. On day 10, mice were randomly divided into each group (*n* = 6 per group) based on body weight. Therefore, there was no difference in body weight before exosome treatment among experimental groups. The sample size was decided based on previous experiences to obtain meaningful data and the availability of mouse facility, and there were no exclusions.

For the single-injection experiment, mice were randomly divided into control exosome generated from un-engineered wild-type Expi293F cells (Exo-Naïve)- or Exo-srIκB-injected group (*n* = 6 per group) on day 10, and exosomes (5.0 × 10^10^ particles per mouse) were intravenously administered via tail vein. After 1 h of exosome injection, 4 g kg^−1^ of 40% EtOH was orally delivered to each mouse and they were sacrificed after 6 h of EtOH gavage [23]. For experiments of 3 consecutive days of injection, mice were randomly assigned in Exo-Naïve (10^9^ particle per mouse)-, low doses of Exo-srIκB (10^8^ particles per mouse)-, or high doses of Exo-srIκB (10^9^ particles per mouse)-injected group (*n* = 6 per group) on day 10. Indicated doses of exosomes were intravenously delivered for 3 consecutive days with 24-hour intervals. After 6 h of last exosome injection, 4 g kg^−1^ of 40% EtOH was orally administered to each mouse. Doses of exosomes used in the present study were determined based on the previous experiments [19,20,21]. Since many hepatic cells underwent apoptosis after acute high doses of EtOH gavage and caused injury, we isolated hepatic cells after 1 h of EtOH gavage for analysis. However, mice were sacrificed after 6 h of EtOH gavage similar to single-injection experiment. Researchers were not blinded for the animal experiments.

### 2.2. Exosomes Production

The exosome production process was previously described [19,24]. In brief, Expi293F producing cells were incubated for 4 days in a wave culture system, and cells were exposed to blue-light illumination for target protein loading and exosome production. After that, the culture medium was harvested and centrifuged at 2000× *g* for 10 min to remove cells and debris. A 0.22 µm of polyethersulfone filter was used to remove large particles. Next, the exosome was purified through the ultrafiltration and diafiltration process for the concentrated harvested culture medium and buffer exchange. Then, exosomes were purified through anionic and multi-modal resin chromatography. Finally, a formulation and sterilization filter process was performed.

### 2.3. Transmission Electron Microscopy

The morphology of extracellular vesicles (EVs) was characterized by transmission electron microscope (TEM). First, EVs were allowed to absorb onto carbon-coated copper grids (Electron Microscopy Sciences, Hatfield, PA, USA) for 15 sec. After removing excess liquid, samples were negatively stained with 2% uranyl acetate (Electron Microscopy Sciences, Hatfield, PA, USA). TEM images were obtained with a Tecnai G2 Retrofit electron microscope (FEI Company, Hillsboro, OR, USA) operating at 200 kV.

### 2.4. Nanoparticle Tracking Analysis

Particle number and size distribution of EVs were measured by nanoparticle tracking analysis (NTA) using NS300 (Malvern Panalytical a spectris company, Malvern, UK). According to the manufacturer’s manual, samples were diluted (1:100 to 1:10,000) in particle-free saline to an acceptable concentration. Samples were analyzed under constant flow conditions at 25 °C, a camera level of 15, and detect threshold of 3. Concentrations of EVs were measured on the basis of counts of 20 to 100 particles per frame.

### 2.5. Next-Generation Sequencing Analyses

Single-cell RNA sequencing (scRNA-seq) analysis of normal mouse liver tissues [25] can be explored on the Tabula Muris Senis website (https://tabula-muris-senis.ds.czbiohub.org/ (accessed on 12 December 2022)) or found in NCBI Gene Expression Omnibus under accession number GSE132042. The bulk RNA-seq of a vehicle or LPS-treated mouse primary KCs [26] is publicly available in NCBI Gene Expression Omnibus under accession number GSE86397. Kyoto encyclopedia of genes and genomes (KEGG) pathway and gene ontology analyses were performed with Database for Annotation, Visualization and Integrated Discovery (DAVID) (https://david.ncifcrf.gov/ (accessed on 21 December 2022).) [27]. scRNA-seq analysis of human liver specimens [28] is publicly available in NCBI Gene Expression Omnibus under accession number GSE136103.

### 2.6. Isolation of Mouse Primary Hepatic Cells

HEPs, hepatic stellate cells (HSCs), KCs, and liver mononuclear cells (MNCs) were isolated from C57BL/6J WT male mice by differential centrifugation on an Opti-Prep (Sigma-Aldrich, St. Louis, MO, USA) or Percoll (Sigma-Aldrich, St. Louis, MO, USA) as previously described [29]. In brief, a two-step collagenase liver perfusion was performed by portal vein cannulation. The liver was first perfused with EGTA solution (5.4 mM KCl, 0.44 mM KH_2_PO_4_, 140 mM NaCl, 0.34 mM Na_2_HPO_4_, 0.5 mM EGTA, 25 mM Tricine, and pH 7.2), followed by the collagenase solution (0.075% collagenase type I (Worthington, Columbus, OH, USA) in Hank’s balanced salt solution (HBSS) with 0.02% DNase I). After complete circulation, the liver was extracted and further digested in the digestion buffer (0.009% of collagenase type I in HBSS with 0.02% of DNase I) at 37 °C in a shaking incubator (90 rpm, 20 min). The digested liver was then filtered through a 70 μm cell strainer to remove undigested debris and connective tissues. To isolate HEP, filtered cell suspension was centrifuged at 50× *g* for 5 min and further purified by 50% Percoll gradient solution. To isolate hepatic non-parenchymal cells, the supernatant from the first centrifugation was obtained and centrifuged at 650× *g* for 10 min. From the whole non-parenchymal cell pellet, HSCs and KCs were isolated by centrifugation at 1800× *g*, 4 °C, for 17 min with 11.5 % and 20 % Opti-prep gradient solution, respectively. Cells obtained were further washed in HBSS before use. For isolation of liver MNCs, the non-parenchymal cell pellet was suspended in 40% Percoll gradient solution and centrifuged at 1800× *g* for 20 min. The supernatant, containing debris, was carefully removed and liver MNCs were resuspended in saline following the lysis of red blood cells.

### 2.7. DiI Labeling of Exo-srIκB

Isolated and purified Exo-srIκB (1.0 × 10^12^ particles) was incubated with 10 μL of DiI at 37 °C for 30 min protected from light. After that, 1 × filtered phosphate-buffered saline was added to up to 4 mL and moved to Amicon^®^ Ultra-4 centrifugal filter unit (Merck, Rahway, NJ, USA). Solutions were eluted by centrifugation at 3200× *g*, 4 °C, for 15 min until the residual volume was approximately 200 μL. Exo-srIκB was directly treated to the culture media of KCs.

### 2.8. Quantitative Polymerase Chain Reaction

Total RNA was extracted from liver tissues or isolated hepatic cells using TRIzol^®^ (Thermo Fisher Scientific, Waltham, MA, USA) and NanoDrop™ Lite (Thermo Fisher Scientific, Waltham, MA, USA) was used to measure the quantity and quality of isolated RNA. A sample had A260/A280 ratio over 1.8 which was used. The cDNA then synthesized from the extracted RNA with ReverTra Ace^®^ qPCR RT Master Mix with gDNA Remover (Toyobo, Osaka, Japan). A quantitative real-time polymerase chain reaction (qRT-PCR) was performed with SYBR Green Real-time PCR Master Mix (Toyobo, Osaka, Japan). All the samples were duplicated in qRT-PCR analyses. The mRNA expression levels of the genes of interest were normalized by *18s* rRNA expression levels. The primer sequences used in qRT-PCR analyses are listed in Table S1.

### 2.9. Histological Analyses

For consistency of sample comparison, similar regions of the left and medial lobes of mouse liver were used for histological analyses. Liver tissues were fixed with 10% neutral buffer formalin (Sigma-Aldrich, St. Louis, MO, USA) overnight at room temperature. After deparaffinization and rehydration, the paraffin-embedded tissues were sliced at 4 μm thickness and stained with hematoxylin (Sigma-Aldrich, St. Louis, MO, USA) and eosin (Sigma-Aldrich, St. Louis, MO, USA) solution. For Oil Red O staining, formalin-fixed liver tissues were embedded in frozen section compound (Leica Biosystems, Wetzlar, DE) and sliced at 10 μm thickness before use. Stained images were captured using light microscopy (Olympus BX51) and analyzed with DP2-BSW software.

Paraffin-embedded liver tissues sectioned at 4 μm thickness were used for immunostaining. Following deparaffinization and rehydration, tissue samples were immersed in 10 mM citrate buffer (pH 6.0) and microwaved for 5 min for antigen retrieval. Tissues were first blocked with 10% donkey serum for 1 h at room temperature, followed by an overnight incubation with C-type lectin domain family 4 member F (CLEC4F) (R&D Systems, MN, USA) or myeloperoxidase (MPO) (Abcam, Cambridge, UK) antibodies (1:50 to 1:200 in saline containing 0.1% Tween-20) at 4 °C. For CLEC4F immunofluorescence staining, sections were incubated with Alexa Fluor^®^ 594-conjugated secondary antibodies (Jackson ImmunoResearch, West Grove, PA, USA) for 1 h at room temperature and covered with DAPI mounting solution (Abcam, Cambridge, UK). For MPO immunohistochemistry analysis, samples were incubated with either anti-Rabbit IgG (Vector Laboratories, Newark, CA, USA) or for 1 h at room temperature and developed with 3.3′-diaminobenzidine (DAB) substrate kit (Vector Laboratories, Newark, CA, USA), followed by Balsam (Sigma-Aldrich, St. Louis, MO, USA) covering. The terminal deoxynucleotidyl transferase dUTP nick end labeling (TUNEL) assay (Abcam, Cambridge, UK) was performed according to the manufacturer’s instructions. Images were captured using Olympus BX51 microscope equipped with a CCD camera (Olympus, Tokyo, Japan) and analyzed with DP2-BSW.

### 2.10. Biochemical Analysis

For hepatic triglyceride (TG) measurement, hepatic lipids were extracted from about 20 to 30 mg of frozen liver tissues using chloroform/methanol (2:1 ratio) solution as previously described [30]. Lyophilized hepatic lipids were then resuspended to 5% bovine serum albumin (BSA)-saline. The VetTest Chemistry analyzer (IDEXX Laboratories, Westbrook, ME, USA) was used to measure the hepatic TG levels and serum levels of alanine aminotransferase (ALT), aspartate aminotransferase (AST), TG, and total cholesterol (TC), according to the manufacturer’s protocols.

### 2.11. Western Blotting

Expi293F producing cells were lysed in RIPA buffer containing Halt™ Protease and Phosphatase Inhibitor Cocktail (100×) (Thermo Fisher Scientific, Waltham, MA, USA). Lysates were centrifuged (12,000 rpm) at 4 °C for 20 min, and the supernatant was used for immunoblotting. Exosome was lysed in 4X Laemmli sample buffer (Bio-RAD, Hercules, CA, USA). Cell lysate protein and exosomes were boiled for 5 min at 100 °C. Samples were run in 10% SDS/polyacrylamide gel electrophoresis (PAGE) gel and transferred onto nitrocellulose membrane. The membranes were blocked by 5% skim milk in Tris-buffered saline containing 0.1% Tween-20 (TBS-T) for 1 h at RT. The membrane was incubated with primary antibodies against srIκB, CRY2 (customized antibody, Abclon, Seoul, Korea), CD9, CD81 (SBI, Tokyo, Japan), TSG101, alix, GM130, calnexin (Abcam, Cambridge, UK), Lamin B1, glyceraldehyde-3-phosphate dehydrogenase (GAPDH) (Santa Cruz Biotechnology, Dallas, TX, USA), and prohibitin (NOVUSBIO, Centennial, CO, USA) at 4 °C overnight. After incubation with specific secondary antibodies, blots were developed using Clarity and Clarity Max ECL Western Blotting Substrates and imaged with the ChemiDoc imager (Bio-Rad, Hercules, CA, USA).

Frozen liver tissues were first subjected to nuclear extraction using the nuclear and cytoplasmic extraction kit (Thermo Fisher Scientific, Waltham, MA, USA). Proteins extracted from the nuclear compartments (to 30 μg) were first separated in a 10% SDS-PAGE, and then transferred onto a nitrocellulose membrane. After the transfer, the membrane was blocked with 5% skim milk solution for 1 h at room temperature, followed by an overnight incubation with NF-κB or lamin B1 (Abcam, Cambridge, UK) antibodies (1:1000 to 1:2000 in saline containing 0.1% Tween-20) at 4 °C. On the next day, membranes were washed and incubated with anti-rabbit secondary antibodies (Cell Signaling Technology, Danvers, MA, USA) for 1 hour at room temperature. Immunoreactive bands were detected by SuperSignal™ West Femto PLUS Chemiluminescent substrate (Thermo Fisher Scientific, Waltham, MA, USA) and captured by ImageQuant™ LAS 4000 (GE Healthcare, Chicago, IL, USA). Nuclear protein levels were normalized to the expression of lamin B1 for each sample. Densitometry analysis was performed with ImageJ (National Institute of Health, Bethesda, MD, USA).

### 2.12. Flow Cytometry Analyses

Isolated liver MNCs were stained with Live/Dead Fixable Aqua Dead Cell Stain Kit (Invitrogen, MA, Waltham, USA) and incubated at room temperature for 15 min protected from light. After washing with flow cytometry buffer solution (0.5% BSA, 0.955% Dulbecco’s phosphate buffered saline, and 0.05% sodium azide) by centrifugation at 350× *g*, 4 °C, for 5 min, samples were labeled with anti-mouse CD16/CD232 (BD Biosciences, Franklin Lakes, NJ, USA) and fluorescence-conjugated eFluor450 anti-mouse CD45 (Invitrogen, Waltham, MA, USA), peridinin-chlorophyll-protein (PerCP)-cyanine (Cy)5.5 anti-mouse CD45 (BD Biosciences, Franklin Lakes, NJ, USA), brilliant violet (BV)786 anti-mouse CD3e (BD Biosciences, Franklin Lakes, NJ, USA), PerCP-Cy5.5 anti-mouse CD4 (BD Biosciences, Franklin Lakes, NJ, USA), allophycocyanin (APC) anti-mouse CD8a (BD Biosciences, Franklin Lakes, NJ, USA), phycoerythrin (PE) anti-mouse NK1.1 (BD Biosciences, Franklin Lakes, NJ, USA), fluorescein isothiocyanate (FITC) anti-mouse F4/80 (Invitrogen, Waltham, MA, USA), PE-Cy7 anti-mouse F4/80 (Invitrogen, Waltham, MA, USA), PE anti-mouse Ly6C (BD Biosciences, Franklin Lakes, NJ, USA), PerCP-Cy5.5 anti-mouse Ly6G (BD Biosciences, Franklin Lakes, NJ, USA), APC-Cy7 anti-mouse CD11b (BD Biosciences, Franklin Lakes, NJ, USA), PE-CF594 anti-mouse Siglec-F (BD Biosciences, Franklin Lakes, NJ, USA), PE-CF594 anti-mouse Ly6G (BD Biosciences, Franklin Lakes, NJ, USA), PE anti-mouse C-type lectin-like receptor 2 (CLEC2) (BioLegend, San Diego, CA, USA), Alexa Fluor^®^ 647 anti-mouse T-cell immunoglobulin mucin-4 (TIM4) (BioLegend, San Diego, CA, USA), and PE-Cy7 anti-mouse CD146 (BioLegend, San Diego, CA, USA) antibodies. After washing with flow cytometry buffer solution by centrifugation at 350× *g*, 4 °C, for 5 min, stained cells were read with LSRFortessa™ X-20 (BD Biosciences, Franklin Lakes, NJ, USA) and analyzed with FlowJo™ v10 software (BD Biosciences, Franklin Lakes, NJ, USA).

### 2.13. Enzyme-Linked Immunosorbent Assay (ELISA)

Mouse IL-6 (#M6000B), IL-1β (#MLB00C), and TNF-α (#MTA00B) Quantikine ELISA kits (R&D Systems, Minneapolis, MN, USA) were used according to the manufacturer’s instructions. Microplates were read by an iMarkTM microplate absorbance reader (Bio-Rad, CA, USA).

### 2.14. Statistical Analyses

All statistical analyses in this study were conducted with Prism version 8.0 (GraphPad Software, San Diego, CA, USA). Statistical significance was examined by a two-tailed Student’s *t*-test between the two groups or a one-way analysis of variance (ANOVA) with Tukey’s multiple comparisons test among three or more groups. For RNA-seq, an adjusted *p* value was used. *p* < 0.05 was thought to be statistically meaningful.

## 3. Results

### 3.1. Characterization and Analysis of Exo-srIκB

**Figure 1:**
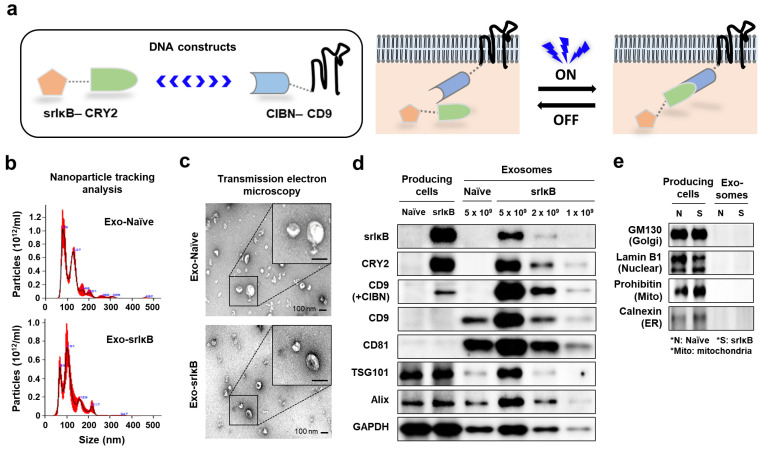
Generation and characterization of Exo-srIκB. (**a**) Schematic diagrams for DNA constructs (**left**) and the blue-light-mediated fusion of recombinant proteins (**right**) in producing Exo-srIκB. (**b**) Representative panels for nanoparticle tracking analysis. (**c**) Representative images for transmission electron microscopy of Exo-Naïve and Exo-srIκB. (**d**,**e**) Western blot analysis of Expi293F cells (producing cells) and Expi293F cell-derived exosomes to analyze the expression of recombinant proteins (srIκB and CD9) and exosome markers (CD9, CD81, TSG101, Alix, and GAPDH) (**d**), and cell organelle markers (GM130, lamin B1, prohibitin, and calnexin) (**e**). CIBN, N-terminal fragment of cryptochrome-interacting basic-helix-loop-helix 1; Exo-srIκB, srIκB-loaded exosomes; GAPDH, glyceraldehyde-3-phosphate dehydrogenase.

We produced and purified exosomes as previously reported [19,24]. The Expi293F cell lines with two recombinant proteins, srIκB-CRY2 and N-terminal fragment of cryptochrome-interacting basic-helix-loop-helix 1 (CIBN)-CD9, were used to produce Exo-srIκB. In particular, we used blue-light illumination to transiently combine CRY2 and CIBN (Figure 1a). The diameter of isolated exosomes was measured by NTA, and it was ranged from 50 to 130 nm as consistent with the reported size of exosomes (Figure 1b) [19]. In addition, TEM confirmed the size and shape of isolated exosomes (Figure 1c). Western blot analysis further identified the protein expression of srIκB, CRY2, and CIBN only in Exo-srIκB, but not in Exo-Naïve, while both exosomes expressed classical exosome markers, such as CD9, CD81, TSG101, Alix, and GAPDH (Figure 1d). In addition, we did not detect the expression of cell organelle marker proteins, including GM130, lamin B1, prohibitin, and calnexin, in both exosomes (Figure 1e).

### 3.2. Expression Levels of Exosome Uptake Process-Related Genes Are Increased in Mice and Human Inflammatory KCs

**Figure 2:**
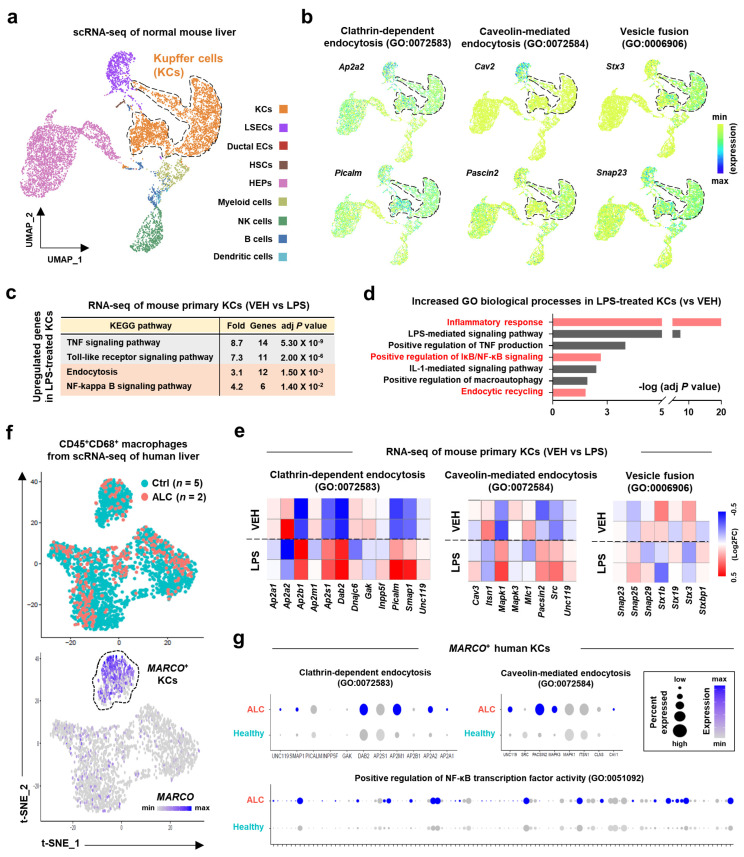
Increased expression levels of exosome uptake process-related genes in mice and human inflammatory Kupffer cells (KCs). (**a**,**b**) Single-cell RNA sequencing (scRNA-seq) of normal mouse liver (GSE132042) was analyzed. Uniform manifold approximation and projection (UMAP) presentation of feature plots for cell type annotation (**a**) or gene expression levels (**b**). (**c**–**e**) Bulk RNA-seq of mouse primary KCs (GSE86397) was analyzed. (**c**) Kyoto encyclopedia of genes and genomes (KEGG) pathway analysis of upregulated genes in lipopolysaccharide (LPS)-treated KCs. (**d**) Increased gene ontology (GO) biological processes in LPS-treated KCs were analyzed. (**e**) Heatmaps showed relative expression levels of genes related to indicated pathways. (**f**,**g**) scRNA-seq of the human liver (GSE136103) from healthy control (Ctrl, *n* = 5) or patients with alcohol-associated liver cirrhosis (ALC, *n* = 2) was analyzed. (**f**) A t-distributed stochastic neighbor embedding (t-SNE) presentation of feature plots showed annotation by group (**upper**) and expression level of *MARCO* (**lower**). (**g**) Relative expression levels of genes related to indicated pathways were analyzed in *MARCO*^+^ KCs. For RNA-seq, an adjusted *p* value was used.

The liver is a major organ where Exo-srIκB is delivered after intravenous injection [22]. Since exosome-mediated regulation of cellular responses of target cells is usually induced by endocytosis or membrane fusion [18,31], we next examined the transcriptional profiles of diverse hepatic cells to investigate the major target cell types for Exo-srIκB. When we analyzed the scRNA-seq of normal mouse liver [32], a total of nine clusters were annotated as KCs, liver sinusoidal endothelial cells (LSECs), ductal epithelial cells, HSCs, HEPs, myeloid cells, natural killer (NK) cells, B cells, and dendritic cells (Figure 2a). We discovered that the expression levels of genes related to exosome uptake processes, such as clathrin-dependent endocytosis (*Ap2a2* and *Picalm*), caveolin-mediated endocytosis (*Cav2* and *Pascin2*), and vesicle fusion (*Stx3* and *Snap23*), were enriched in LSECs or KCs (Figure 2b). Due to the anatomical location of KCs over the LSECs, it might be plausible that KCs might primarily take up Exo-srIκB after intravenous injection. Next, we explored whether inflammatory activation of KCs could affect the expression levels of genes related to exosome uptake processes. Interestingly, bulk RNA-seq analysis of vehicle (VEH)- or LPS-treated mouse primary KCs [26] revealed that expression levels of genes related to clathrin-dependent or caveolin-mediated endocytosis pathways, but not vesicle fusion process, was elevated along with inflammatory responses and NF-κB signaling pathways in LPS-treated KCs compared to those of VEH-treated KCs (Figure 2c–e). Consistently, expression levels of genes related to clathrin-dependent endocytosis, caveolin-mediated endocytosis, and positive regulation of NF-κB transcription factor activity were upregulated in scRNA-seq of *MARCO*^+^ human KCs isolated from patients with ALC compared to those of healthy controls (Ctrl) (Figure 2f,g). These data suggest that drug-loaded exosomes might be efficiently delivered to activated KCs and exert its effect.

### 3.3. Exo-srIκB Reduces Inflammatory Gene Expression Levels of LPS-Treated Mouse KCs In Vitro

**Figure 3:**
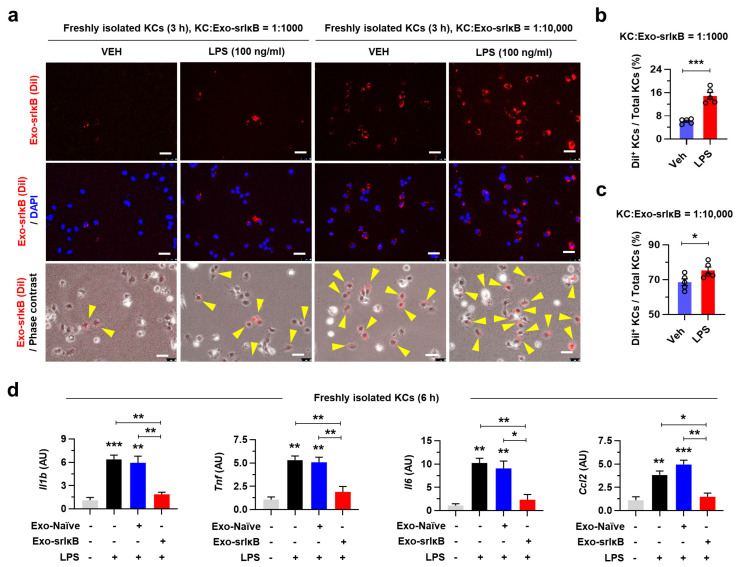
Exo-srIκB suppresses LPS-induced inflammatory gene expression levels in isolated mouse KCs. (**a**–**c**) Indicated doses of DiI-labeled Exo-srIκB were treated to isolated mouse KCs with vehicle (saline, VEH) or LPS (100 ng mL^−1^) for 3 h (*n* = 5 per group). (**a**) Representative pictures of cultured mouse KCs. Scale bars, 50 µm. (**b**,**c**) The frequencies of DiI^+^ KCs among total KCs. (**d**) Quantitative real-time polymerase chain reaction (qRT-PCR) analyses of isolated mouse KCs pretreated with control exosomes (Exo-Naïve) or Exo-srIκB (KC:exosome = 1:10,000) for 3 h followed by LPS (100 ng mL^−1^) for 6 h (*n* = 3 per group). Data are presented as mean ± SEM. * *p* < 0.05, ** *p* < 0.01, *** *p* < 0.001. Data were analyzed by a two-tailed Student’s *t*-test or one-way analysis of variance (ANOVA) with Tukey’s multiple comparisons test.

We next tried to demonstrate the efficient delivery of Exo-srIκB in KCs and its inhibitory effect on LPS-induced inflammatory gene expression levels in vitro. First, to investigate the delivery of Exo-srIκB by KCs, we labeled Exo-srIκB with DiI and treated to mouse primary KCs in time (1 or 3 h)- and dose (KC:Exo-srIκB = 1:1000 or 1:10,000)-dependent manners. Although the Exo-srIκB-laden KCs could not be detected after 1 h (Figure S1a), Exo-srIκB was delivered to about 8% of KCs at low dose (KC:Exo-srIκB = 1:1000) and 70% at high dose (KC:Exo-srIκB = 1:10,000) after 3 h of treatment (Figure 3a-c). In addition, in line with the findings in Figure 2, LPS treatment significantly enhanced the uptake of Exo-srIκB by KCs (Figure 3a–c). To explore the suppressive role of Exo-srIκB on LPS-induced inflammatory activation in KCs, we pretreated Exo-Naïve or Exo-srIκB to isolated mouse KCs followed by LPS. The qRT-PCR analyses revealed that treatment of Exo-srIκB, but not Exo-Naïve, normalized the increased expression levels of inflammatory genes (Il1b, Tnf, Il6, and Ccl2) by LPS without inducing cell death (Figure 3d and Figure S1b).

### 3.4. A Single Administration of Exo-srIκB Ameliorates AFL but Not ALI in Mice

**Figure 4:**
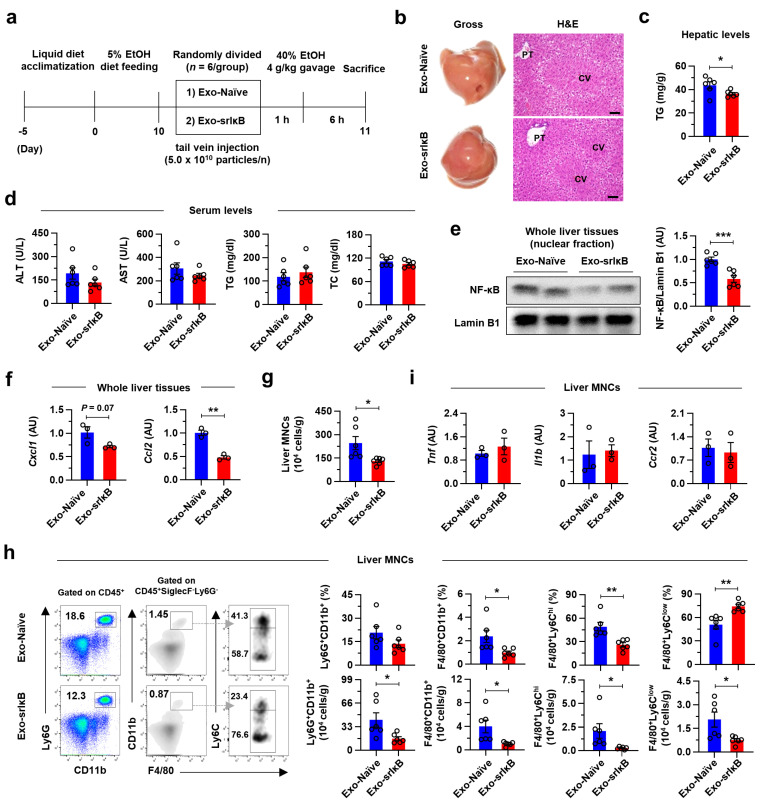
A single high dose of Exo-srIκB ameliorates AFL but not ALI in mice. (**a**–**h**) Wild-type (WT) male mice were fed ethanol (EtOH) for 10 days and randomly divided into Exo-Naïve or Exo-srIκB groups (*n* = 6 per group). A single high dose (5.0 × 10^10^ particles per mouse) of exosomes were intravenously injected. After 1 h, mice were given acute EtOH binge drinking (4 g kg^−1^ of 40% EtOH gavage) and sacrificed after 6 h. (**a**) Schematic diagram of experiments. (**b**) Representative gross finding and H&E staining of liver tissues. Central vein (CV) and portal triad (PT). Scale bars, 50 µm. (**c**) Hepatic triglyceride (TG) levels were measured. (**d**) Serum levels of alanine aminotransferase (ALT), aspartate aminotransferase (AST), TG, and total cholesterol (TC) were measured. (**e**) Representative Western blot analyses of hepatic nuclear fraction (two blots/group) and densitometry analysis (*n* = 6 per group). (**f**) qRT-PCR analyses of whole liver tissues (*n* = 3 per group). (**g**) The number of mononuclear cells (MNCs) per gram of liver tissue. (**h**) Flow cytometry analyses of liver MNCs with representative panels and bar graphs indicating frequencies or absolute numbers of the indicated population. (**i**) qRT-PCR analyses of liver MNCs (*n* = 3 per group). Data are presented as mean ± SEM. * *p* < 0.05, ** *p* < 0.01, *** *p* < 0.001. Data were analyzed by a two-tailed Student’s *t*-test.

Prompted by the above findings from in vitro experiments, we explored the therapeutic potential of Exo-srIκB in an acute-on-chronic ALI experimental model [23]. After 10 days of chronic EtOH feeding, we randomly divided mice into 2 groups and intravenously injected a single high dose (5.0 *×* 10^10^ particles per mouse) of Exo-Naïve or Exo-srIκB before acute binge drinking (Figure 4a). In H&E staining of liver tissues, we found a significant decrease in AFL in Exo-srIκB-treated mice compared to that of Exo-Naïve-treated mice as confirmed by hepatic TG measurement (Figure 4b,c). However, although serum levels of ALT and AST tended to reduce by Exo-srIκB, there were no differences in serum TG and TC levels and body weight between the two groups (Figure 4d and Figure S2a). To confirm the hepatic delivery of Exo-srIκB, we measured NF-κB levels in the nuclear fraction of whole liver tissues by Western blot and notably decreased nuclear translocation of NF-κB was detected in Exo-srIκB-treated mice compared to that of control mice (Figure 4e). A previous report has demonstrated that NF-κB regulates the transcription of Cxcl1 and Ccl2, chemokine ligands for neutrophil or monocyte recruitment, respectively [4]. Consistently, Exo-srIκB treatment suppressed hepatic expression levels of Cxcl1 and Ccl2, resulting in a lower number of MNCs per gram of liver tissue in Exo-srIκB-treated mice compared to that of the control mice (Figure 4f,g). Flow cytometry analyses further revealed that hepatic frequencies of F4/80^+^CD11b^+^ infiltrating macrophages, especially F4/80^+^Ly6C^hi^ pro-inflammatory macrophages, were decreased by Exo-srIκB treatment, whereas the frequencies of F4/80^+^Ly6C^low^ anti-inflammatory macrophages were increased in Exo-srIκB group and Ly6G^+^CD11b^+^ neutrophils or lymphocytes were similar between the two groups (Figure 4h and Figure S2b). When we measured the absolute numbers of each population per gram of liver tissue, all the above cell types showed significantly diminished numbers except NK and NKT cells after Exo-srIκB treatment (Figure 4h and Figure S2b). However, unexpectedly, expression levels of inflammatory genes (Tnf, Il1b, and Ccr2) in the liver MNCs were similar between the two groups (Figure 4i). These results imply that a single dose of Exo-srIκB, although protective, is insufficient to avert alcohol-associated liver damage and inflammation caused by acute binge drinking.

### 3.5. Three Consecutive Days of Exo-srIκB Injection Mitigates ALI, AFL, and ALF in Mice

**Figure 5:**
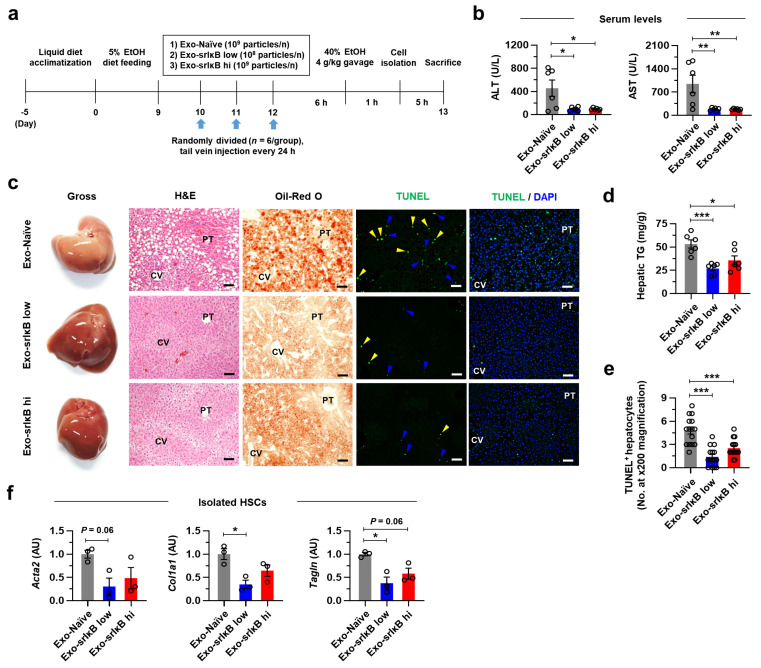
Three consecutive days of Exo-srIκB injection mitigates ALI, AFL, and ALF in mice. (**a**–**f**) WT male mice were fed EtOH for nine days and randomly divided into Exo-Naïve (10^9^ particles/day/mouse), low dose of Exo-srIκB (10^8^ particles/day/mouse), or high dose of Exo-srIκB (10^9^ particles/day/mouse) groups (*n* = 6 per group). An indicated dose of exosomes was intravenously injected every 24 hours for consecutive 3 days. After 6 h of the last injection, mice were given acute EtOH binge drinking (4 g kg^−1^ of 40% EtOH gavage) and sacrificed after 6 h. (**a**) Schematic diagram of experiments. (**b**) Serum levels of ALT and AST were measured. (**c**) Representative gross finding, H&E, Oil Red O, and terminal deoxynucleotidyl transferase dUTP nick end labeling (TUNEL) staining of liver tissues. Scale bars, 50 µm. In TUNEL staining, apoptotic hepatocytes (HEPs) (yellow triangles) and non-parenchymal cells (blue triangles) were indicated. (**d**) Hepatic TG levels were measured. (**e**) TUNEL^+^ apoptotic HEPs were counted in 15 random images at ×100 magnification. (**f**) qRT-PCR analyses of isolated hepatic stellate cells (HSCs) (*n* = 3 per group). Data are presented as mean ± SEM. * *p* < 0.05, ** *p* < 0.01, *** *p* < 0.001. Data were analyzed by a one-way ANOVA with Tukey’s multiple comparisons test.

We next examined the preventative effects of multiple Exo-srIκB treatments on ALI. To do this, we fed EtOH to WT mice for 10 days and randomly divided them into 3 groups, which received 3 consecutive days of Exo-Naïve (10^9^ particles/day/mouse), low dose of Exo-srIκB (10^8^ particles/day/mouse), or high dose of Exo-srIκB (10^9^ particles/day/mouse) before alcohol binge drinking (Figure 5a). At sacrifice, we found significantly reduced serum ALT and AST levels in both low-dose and high-dose Exo-srIκB injected mice compared to those of control mice with similar body weight and serum TG and TC levels (Figure 5b and Figure S3a–c). In addition, gross findings, H&E staining, and Oil Red O staining of liver tissues identified that lipid accumulation in the midzonal HEPs was markedly suppressed by Exo-srIκB treatment regardless of dosage as confirmed by hepatic TG measurement (Figure 5c,d). Moreover, TUNEL assay of liver tissue showed that Exo-srIκB treatment diminished apoptosis of HEPs and non-parenchymal cells in the midzonal area of Exo-Naïve-treated mice (Figure 5c,d). Finally, in isolated HSCs, expression levels of genes related to fibrotic activation (Acta2, Col1a1, and Tagln) were significantly reduced in the low-dose Exo-srIκB group and tended to decrease in the high-dose Exo-srIκB group compared to those of the control group (Figure 5f).

### 3.6. Three Consecutive Days of Exo-srIκB Injection Attenuates Alcohol-Associated Hepatic Inflammation by Suppressing the Activation of KCs in Mice

**Figure 6:**
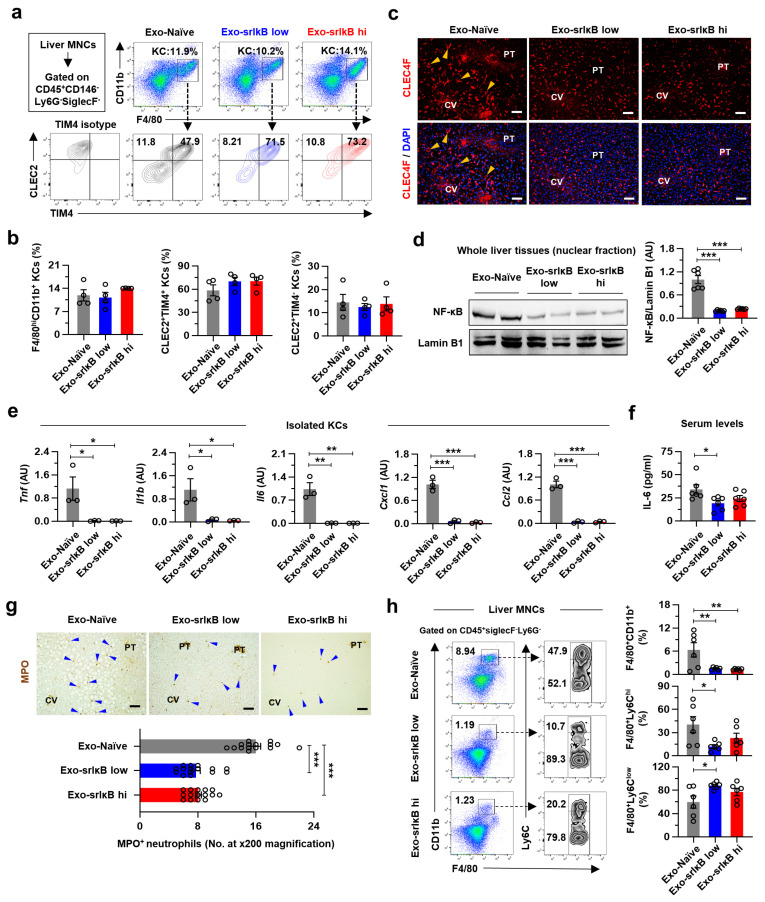
Three consecutive days of Exo-srIκB injection attenuates alcohol-associated hepatic inflammation by suppressing the activation of KCs in mice. (**a**–**h**) WT male mice were fed EtOH for nine days and randomly divided into Exo-Naïve, low dose of Exo-srIκB, or high dose of Exo-srIκB groups (*n* = 6 per group). An indicated dose of exosomes was intravenously injected every 24 hours for consecutive 3 days. After 6 h of the last injection, mice were given acute EtOH binge drinking (4 g kg^−1^ of 40% EtOH gavage) and sacrificed after 6 h. (**a**,**b**) Flow cytometry analyses of F4/80^hi^CD11b^+^ KCs with C-type lectin-like receptor 2 (CLEC2) and T-cell immunoglobulin and mucin domain containing 4 (TIM4). Representative panels (**a**) with bar graphs (**b**) (*n* = 4 per group). (**c**) Representative C-type lectin domain family 4 member F (CLEC4F) immunostaining of liver tissues. Morphological changes in CLEC4F^+^ KCs were indicated by yellow triangles. (**d**) Representative Western blot analyses of hepatic nuclear fraction (two blots/group) and densitometry analysis (*n* = 6 per group). (**e**) qRT-PCR analyses of isolated KCs (*n* = 3 per group). (**f**) Serum IL-6 levels were measured. (**g**) Representative myeloperoxidase (MPO) immunostaining of liver tissues (**upper**). MPO^+^ neutrophils were indicated by blue triangles and counted in 15 random images at ×200 magnification (**lower**). (**h**) Flow cytometry analyses of liver MNCs with representative panels and bar graphs. Data are presented as mean ± SEM. * *p* < 0.05, ** *p* < 0.01, *** *p* < 0.001. Data were analyzed by a one-way ANOVA with Tukey’s multiple comparisons test. Scale bars, 50 µm.

To explore the underlying mechanisms of the protective effects of Exo-srIκB in ALD, we analyzed the KCs, the major target cell type for Exo-srIκB. We first investigated the population changes in KCs since recent studies have demonstrated that in the progression of non-alcoholic steatohepatitis, there is a gradual loss of embryonic-derived KCs (emKCs) and an eventual replacement by bone marrow-derived macrophages (bmKCs) [33,34]. In particular, the above reports used CLEC2 and TIM4 as markers for emKCs (CLEC2^+^TIM4^+^), which can distinguish them from bmKCs (CLEC2^+^TIM4^−^). In flow cytometry analyses, there were no differences in the frequencies of total F4/80^hi^CD11b^+^ KCs, emKCs (CLEC2^+^TIM4^+^), and bmKCs (CLEC2^+^TIM4^−^) between the three groups (Figure 6a,b). Consistently, immunostaining of liver tissues showed a similar number of CLEC4F (a marker for both emKCs and bmKCs)-expressing cells (Figure 6c). However, in the liver tissues of Exo-Naïve-treated mice, morphological changes were found in the midzonal CLEC4F^+^ KCs, which disappeared by Exo-srIκB treatment, suggesting functional alterations in KCs by Exo-srIκB (Figure 6c). Indeed, we found reduced nuclear translocation of NF-κB and expression levels of pro-inflammatory genes (Tnf, Il1b, and Il6) in whole liver tissues or isolated KCs from Exo-srIκB-treated mice compared to that of control mice (Figure 6d,e). These changes were reflected in decreased serum IL-6 levels in Exo-srIκB-treated mice, although serum levels of tumor necrosis factor TNF-α and IL-1β were lower than the detection limits (Figure 6f and Figure S4a). In addition, expression levels of Cxcl1 and Ccl2 in isolated KCs and HEPs from Exo-srIκB-treated mice were decreased compared to those of control mice (Figure 6e and Figure S4b). However, although the number of F4/80^+^CD11b^+^ macrophages, F4/80^+^Ly6C^hi^ pro-inflammatory macrophages, and F4/80^+^Ly6C^low^ anti-inflammatory macrophages tended to decrease by Exo-srIκB treatment by dose, there were no differences in the number of Ly6G^+^CD11b^+^ neutrophils and total isolated MNCs per gram of liver tissues (Figure S4c,d). These results were presumably derived from the loss of highly activated granulocytes during the isolation steps in Exo-Naïve-treated mice, as shown by lower frequencies of live FSC^hi^ cells in Exo-Naïve-treated mice than in Exo-srIκB-treated mice (Figure S4e). In fact, the number of MPO^+^ neutrophils was significantly decreased in Exo-srIκB-treated mice in the intact liver tissue, especially in the midzonal area (Figure 6g). In addition, except for the FSC^hi^ granulocytes such as Ly6G^+^CD11b^+^ neutrophils that showed similar frequencies in all groups, we could detect the diminished hepatic frequencies of F4/80^+^CD11b^+^ macrophages, particularly F4/80^+^Ly6C^hi^ pro-inflammatory macrophages, and increased frequencies of F4/80^+^Ly6C^low^ anti-inflammatory macrophages in Exo-srIκB-treated mice compared to those of the control mice (Figure 6h and Figure S4e). Moreover, unexpectedly, hepatic frequencies of NK cells were reduced by Exo-srIκB treatment while the frequencies of other lymphocytes were similar (Figure S4f). Together, these results indicate the hepatoprotective effects of multiple Exo-srIκB injections in ALD mediated by suppression of inflammatory KCs.

## 4. Discussions

Chronic alcohol consumption results in an upsurge in intestinal permeability, which leads to the hepatic translocation of endotoxins (e.g., LPS) through the portal vein. The continuous influx of LPS instigates alcohol-associated hepatic inflammation by activating TLR4/NF-κB signaling pathways in KCs. In particular, NF-κB activation in KCs promotes the production of inflammatory cytokines (e.g., TNF-α, IL-6, and IL-1β) and chemokines (e.g., CCL2 and CXCL1) to induce death of HEPs and recruitment of inflammatory monocytes and neutrophils [1]. Although there have been many efforts to abate these processes, there is still no approved drug for ALD. Since liver damage could not be reversed in the late stage of ALD such as ALC or HCC [2], there is an urgent need for a new therapeutic agent, especially targeting early-stage ALD. In the present study, we provide several lines of evidence for the efficient preventive effect of Exo-srIκB against ALI, AFL, and ALF in mice. In particular, we reveal that the beneficial effects of Exo-srIκB are mediated primarily by limiting the inflammatory activation of KCs. These findings imply that exosome-based targeting inflammatory KCs may have therapeutic potential in ALD. In addition, our results expand the prior research demonstrating the protective role of Exo-srIκB in the inflammatory responses of sepsis [19], preterm birth [20], and ischemia-reperfusion kidney injury [21], suggesting the possible application of Exo-srIκB to broad inflammatory diseases.

Previous studies with LBP [8], CD14 [9], or TLR4 [10] knockout (KO) mice reported a crucial role of NF-κB signaling pathway in the development of ALD. Interestingly, the ALI is not reduced in MyD88 KO mice, indicating alcohol-specific MyD88-independent activation of NF-κB [35]. However, since the above studies used whole-body KO mice, identifying the cell type for drug targeting is an arduous task. Therefore, despite promising results in animal experiments, there are no clinical trials conducted with NF-κB inhibitors in ALD. We found highly enriched gene expression levels of exosome uptake processes in KCs among various hepatic cells by analyzing scRNA-seq data [32]. In addition, inflammatory gene expression levels were most dramatically suppressed in KCs after three consecutive days of Exo-srIκB injection with attenuated ALI. These data suggest that targeting NF-κB signaling pathways in KCs might be an effective approach in ALD.

KCs are heterogenous populations depending on their origins. Originally, KCs are derived from the yolk sac and reside in the liver sinusoid. However, chronic liver injury in (non-)alcoholic liver diseases triggers apoptosis of emKCs and their replacement by bmKCs, which are primarily regulated by liver X receptor-α signaling pathways [33,34]. Interestingly, we showed that systemic administration of Exo-srIκB prevented the pro-inflammatory activation of KCs without affecting the frequencies of emKCs and bmKCs in mice with ALD. In addition, the morphological changes in CLEC4F^+^ KCs of Exo-Naïve-treated EtOH-fed mice in the midzonal area disappeared by Exo-srIκB treatment. Moreover, all of the pathologic findings, including lipid accumulation, apoptosis, and infiltration of MPO^+^ neutrophils, were found in the hepatic midzonal area of Exo-Naïve-treated mice. These results may indicate that there might be a functional heterogeneity in KCs depending on their locations across the hepatic lobules in ALD; perivenous KCs undergo apoptosis, midzonal KCs provoke inflammation, and periportal KCs limit endotoxin levels by phagocytosis [36,37]. It would be interesting to define the functional and spatial heterogeneity of KCs in detail by utilizing spatial multi-omics technology for more precise targeting of inflammatory KCs in ALD.

Recent advances in nanotechnology enable the development of nanomedicine for small-molecule therapeutics. However, this approach needs a high dose of the drug because of the low target specificity, resulting in severe adverse effects [13]. In our animal experiments, we showed a similar extent of preventative effects in low (10^8^ particles) and high (10^9^ particles) doses of Exo-srIκB against acute-on-chronic ALI without unexpected rise of ALT in serum. These results indicate the high potency and efficiency of Exo-srIκB in ALD, consistent with the previous finding that demonstrates liver-oriented biodistribution of Exo-srIκB [22]. In addition, although the number of analyzed patients was small, we detected enhanced expression levels of genes related to exosome uptake processes in *MARCO*^+^ resident KCs of patients with ALC compared to those of the healthy controls. Based on the above results, favorable outcomes can be expected from clinical trials of Exo-srIκB in patients with ALD.

Our study has some limitations. First, since the exosomes used in the present study originated from human cell lines, further study is needed to elucidate the mechanisms of action of Exo-srIκB in detail to minimize the unexpected adverse effects. In addition, we detected a significant decrease in the hepatic frequencies of NK cells after multiple injections of Exo-srIκB in mice with ALD. NK cells are innate immune cells that play important roles in protecting against viral hepatitis and liver fibrosis by killing virus-infected HEPs or fibrotic HSCs [29,38]. Even though Exo-srIκB treatment decreased expression levels of genes related to fibrotic activation in HSCs, the usage of Exo-srIκB in late-stage ALD should be performed with caution.

In conclusion, the present study demonstrates the protective role of Exo-srIκB in ALI, especially in suppressing inflammatory activation of KCs in the animal model. Our study has a particular significance in that it suggests a novel exosome-based therapeutic paradigm in ALD.

## Data Availability

The datasets analyzed in the present manuscript are publicly available with accession numbers identified in the Materials and Methods or figure legends.

## Supplementary Materials

The following supporting information can be downloaded at: https://www.mdpi.com/article/10.3390/pharmaceutics15020636/s1, Figure S1: Exo-Naïve or Exo-srIκB treatment does not induce cell death of primary mouse Kupffer cells; Figure S2: A single administration of Exo-srIκB does not alter the body weight and hepatic frequencies of lymphocytes in mice with ALD; Figure S3: Three consecutive days of Exo-srIκB injection does not affect body weight and serum TG and TC levels in mice with ALD; Figure S4: Three consecutive days of Exo-srIκB injection tends to reduce the hepatic infiltration of immune cells in mice with ALD; Table S1: Primer sequences for qPCR.
