# Supplementary material for: Exosome-Based Delivery of Super-Repressor IκBα Alleviates Alcohol-Associated Liver Injury in Mice
Source: Pharmaceutics. 2023 Feb 14;15(2):636. doi: 10.3390/pharmaceutics15020636 (PMC9965399; doi:10.3390/pharmaceutics15020636)
Supplement: Supplementary file 1 [file pharmaceutics-15-00636-s001.zip › pharmaceutics-2193848-supplementary.pdf]

1. SUPPLEMENTARY FIGURES

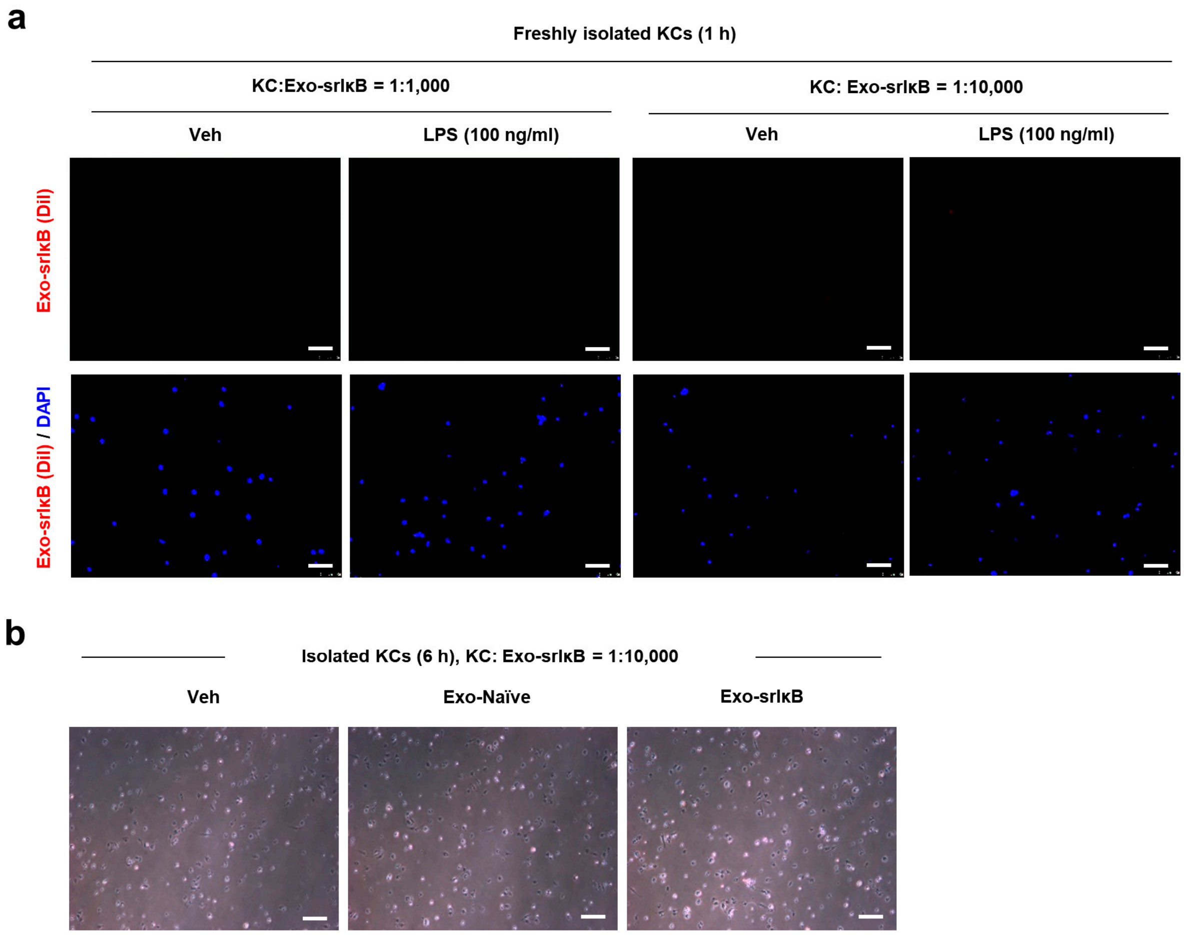

Figure S1. Exo-Naïve or Exo-srIκB treatment does not induce cell death of primary mouse Kupffer cells. (a) Representative pictures for culture plates after indicated doses of DiI-labeled Exo-srIκB treatment to primary mouse KCs with vehicle (saline, VEH) or LPS (100 ng ml^{-1}) for 1 h (n = 5/group). (b) Representative pictures for culture plates after KC:exosomes = 1:10,000 doses of Exo-Naïve or Exo-srIκB treatment to primary mouse KCs with vehicle (saline, VEH) for 6 h (triplicated). Scale bars, 50 μm.

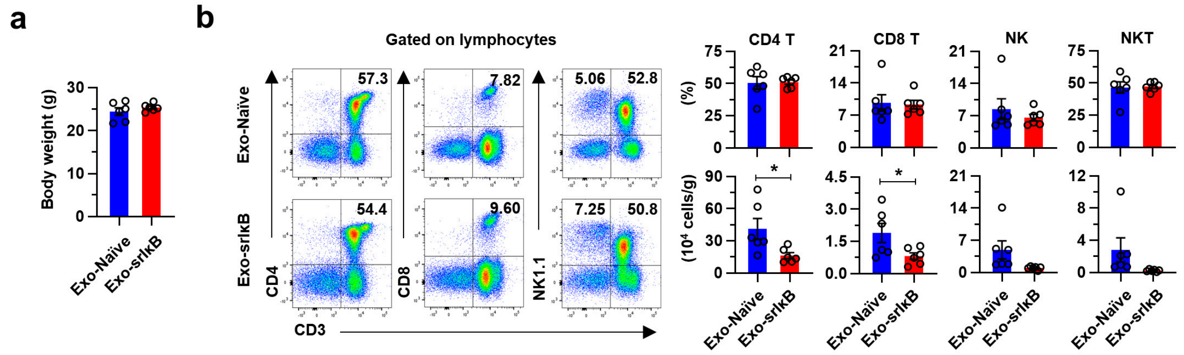

Figure S2. A single administration of Exo-srIκB does not alter the body weight and hepatic frequencies of lymphocytes in mice with ALD. (a,b) WT male mice were fed EtOH for 10 days and randomly divided into Exo-Naïve or Exo-srIκB groups (*n* = 6/group). A single dose (5.0×10^{10} particles per mouse) of exosomes were intravenously injected. After 1 h, mice were given acute EtOH binge drinking (4 g kg^{-1} of 40% EtOH gavage) and sacrificed after 6 h. (a) Body weight was measured at sacrifice. (b) Flow cytometry analyses of hepatic lymphocytes with representative panels and bar graphs indicating frequencies (upper) or absolute number per gram of liver tissues (lower) of lymphocytes. Data are presented as mean ± SEM. *$P < 0.05$. Data were analyzed by a two-tailed Student's *t*-test.

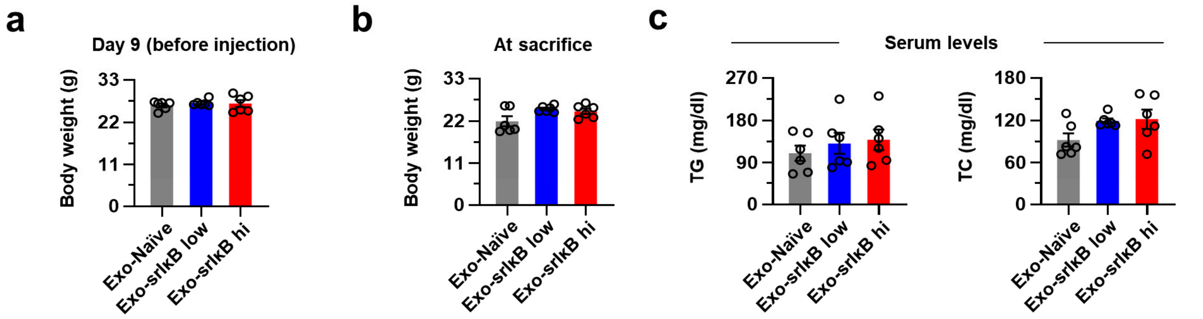

Figure S3. Three consecutive days of Exo-srIκB injection does not affect body weight and serum TG and TC levels in mice with ALD. (a-c) WT male mice were fed EtOH for 9 days and randomly divided into Exo-Naïve (10^9 particles/day/mouse), low-dose of Exo-srIκB (10^8 particles/day/mouse), or high-dose of Exo-srIκB (10^9 particles/day/mouse) groups (*n* = 6/group). An indicated dose of exosomes was intravenously injected every 24 h for consecutive three days. After 6 h of the last injection, mice were given acute EtOH binge drinking (4 g kg^{-1} of 40% EtOH gavage) and sacrificed after 6 h. (a,b) Body weight was measured on day 9 (a) or at sacrifice (b). (c) Serum TG and TC levels were measured. Data are presented as mean ± SEM. Data were analyzed by a One-way ANOVA with Tukey's multiple comparisons test.

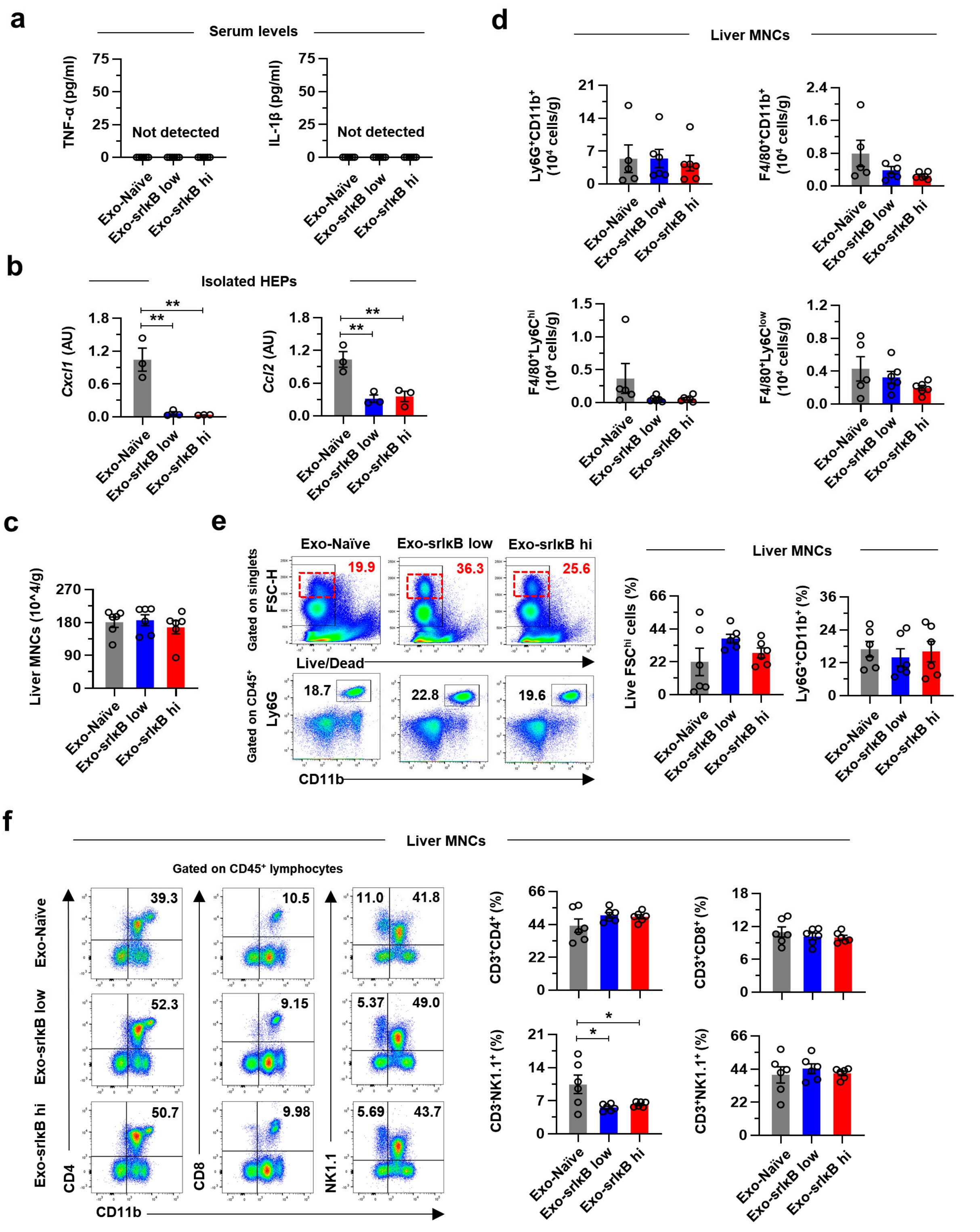

Figure S4. Three consecutive days of Exo-srIκB injection tends to reduce the hepatic infiltration of immune cells in mice with ALD. (a-f) WT male mice were fed EtOH for 9 days and randomly divided into Exo-Naïve (10^9 particles/day/mouse), low-dose of Exo-srIκB (10^8 particles/day/mouse), or high-dose of Exo-srIκB (10^9 particles/day/mouse) groups (n = 6/group). An indicated dose of exosomes was intravenously injected every 24 h for consecutive

three days. After 6 h of the last injection, mice were given acute EtOH binge drinking (4 g kg^{-1} of 40% EtOH gavage) and sacrificed after 6 h. (a) Serum TNF-α and IL-1β levels were measured. (b) qRT-PCR analyses of isolated HEPs (*n* = 3/group). (c) MNC cell counts per gram of liver tissues. (d-f) Flow cytometry analyses. (d) The absolute number of indicated populations per gram of liver tissues. (e) Hepatic frequencies of live FSC^{hi} cells and $Ly6G^{+}CD11b^{+}$ neutrophils with representative panels and bar graphs. (f) Hepatic frequencies of lymphocytes with representative panels and bar graphs. Data are presented as mean ± SEM. $^{*}P < 0.05$, $^{**}P < 0.01$. Data were analyzed by a One-way ANOVA with Tukey's multiple comparisons test.

2. SUPPLEMENTARY TABLE

Table S1. Primer sequences for qPCR.

Genes	Forward	Reverse
18s	ACA GGA TTG ACA GAT TGA TAG C	GCC AGA GTC TCG TTC GTT A
Acta2	GTC CCA GAC ATC AGG GAG TAA	TCG GAT ACT TCA GCG TCA GGA
Ccl2	TTA AAA ACC TGG ATC GGA ACC AA	GCA TTA GCT TCA GAT TTA CGG GT
Col1a1	GCT CCT CTT AGG GGC CAC T	CCA CGT CTC ACC ATT GGG G
Cxcl1	CTG GGA TTC ACC TCA AGA ACA TC	CAG GGT CAA GGC AAG CCT C
Il1b	GCC CAT CCT CTG TGA CTC AT	AGG CCA CAG GTA TTT TGT CG
Il6	TAG TCC TTC CTA CCC CAA TTT CC	TTG GTC CTT AGC CAC TCC TTC
Tagln	CAA CAA GGG TCC ATC CTA CGG	ATC TGG GCG GCC TAC ATC A
Tnf	AAG CCT GTA GCC CAC GTC GTA	AAG GTA CAA CCC ATC GGC TGG
